# Sonographic evaluation of lateral meniscal extrusion: implementation and validation

**DOI:** 10.1007/s00402-020-03683-1

**Published:** 2020-11-20

**Authors:** Philipp W. Winkler, Robert Csapo, Guido Wierer, Caroline Hepperger, Bernhard Heinzle, Andreas B. Imhoff, Christian Hoser, Christian Fink

**Affiliations:** 1 Gelenkpunkt, Sports- and Joint Surgery, Olympiastraße 39, 6020 Innsbruck, Austria; 2 Department for Orthopaedic Sports Medicine, Klinikum rechts der Isar, Technical University of Munich, Ismaninger Str. 22, 81675 Munich, Germany; 3 Research Unit for Orthopaedic Sports Medicine and Injury Prevention, Institute for Sports Medicine, Alpine Medicine and Health Tourism (ISAG), UMIT, Eduard-Wallnöfer-Zentrum 1, 6060 Hall in Tirol, Austria; 4 Department of Orthopedics and Traumatology, Paracelsus Medical University Salzburg, Müllner Hauptstraße 48, 5020 Salzburg, Austria; 5 Department of Radiology, MRT-CT Diagnostics Wörgl, Fritz-Atzl-Str. 8, 6300 Wörgl, Austria

**Keywords:** Lateral meniscal extrusion, Ultrasound, Stress MRI, Validation, Dynamic ultrasound, Dynamic extrusion

## Abstract

**Introduction:**

Meniscal extrusion (ME) is an important indicator of and prognostic factor for various knee pathologies. To date, no standardized protocol for the ultrasound-based examination of lateral ME exists. The purpose of the present study was to test the reliability and validity of lateral ME measurements using a standardized ultrasound-based examination protocol.

**Materials and Methods:**

A group consisting of 11 healthy volunteers (Group I, male and female, 18–45 years) as well as a group of 10 consecutive patients who had undergone all-inside lateral meniscal radial tear repair were included (Group II, male and female, 23–43 years). Lateral ME, the main outcome parameter, was measured by ultrasound (US; both groups) and magnetic resonance imaging (MRI; Group II only). Both knees of all subjects were examined in an unloaded state and under axial compression of the knee (50% of body weight). Repeated measurements obtained in Group I by 2 observers were used for reliability testing, and the validity of US was assessed through comparison with MRI data (Group II).

**Results:**

A total of 66 US images of Group I, obtained by each observer, were analyzed for reliability testing. Forty US and MR images of Group II were assessed for validation. Results showed good interrater (ICC = 0.904) and excellent intrarater (ICC = 0.942) reliability of US-based measurements of lateral ME. Agreement with MRI results was poor (ICC = 0.439), with US systematically overestimating results by 1.1 mm on average.

**Conclusions:**

Ultrasound is a reliable, quick and cost-effective technique for lateral ME measurement, but results are not readily comparable with MRI.

**Trial registration:**

The study was registered in the European Union Clinical Trials Register (EudraCT-Number: 2017-005037-24).

## Introduction

Meniscal extrusion (ME) is defined as the radial displacement of meniscal tissue beyond the tibial margin and is caused by the triangular cross-section of the meniscus in response to axial load application [1]. Physiological extrusion occurs in both the medial [2–6] and lateral [4–7] meniscus. The amount of extrusion is influenced by several patient- and joint-related parameters. Increasing age or body mass index (BMI) [3, 8] as well as the presence of osteoarthritis [2, 5, 9–11] or meniscal tears [7, 8, 12] lead to higher levels of ME. In addition, prolonged periods of meniscus stress may cause a reversible increase in extrusion [13]. Hence, the limits of physiological ME are patient-specific and best determined through comparison with the (healthy) contralateral side, serving as a reference value.

Magnetic resonance imaging (MRI) is considered the gold standard for the assessment of ME [14]. However, MRI is expensive and not always readily available. Also, the acquisition of MR images may be time-consuming and complex. This particularly holds true for stress images, which require the use of special devices for load application [6, 11, 15]. In clinical practice, ME an indicator of various pathologies needs to be recorded quickly, simply and cost-effectively. One technique meeting these requirements is ultrasound (US), which allows for dynamic assessments and has been successfully used for measurements of medial ME in numerous studies [3, 10, 16–19].

The purpose of the present study was to determine the validity and reliability of US for ME measurements of the lateral meniscus under different loading conditions. Therefore, US images of the postero-lateral meniscus corner were acquired using a standardized protocol. Measurements of lateral ME were compared to MRI recordings and tested for intra- and interrater reliability. It was hypothesized that US-based measurements of lateral ME would be reliable and show results that are comparable to those obtained with MRI.

## Methods

**Fig. 1:**
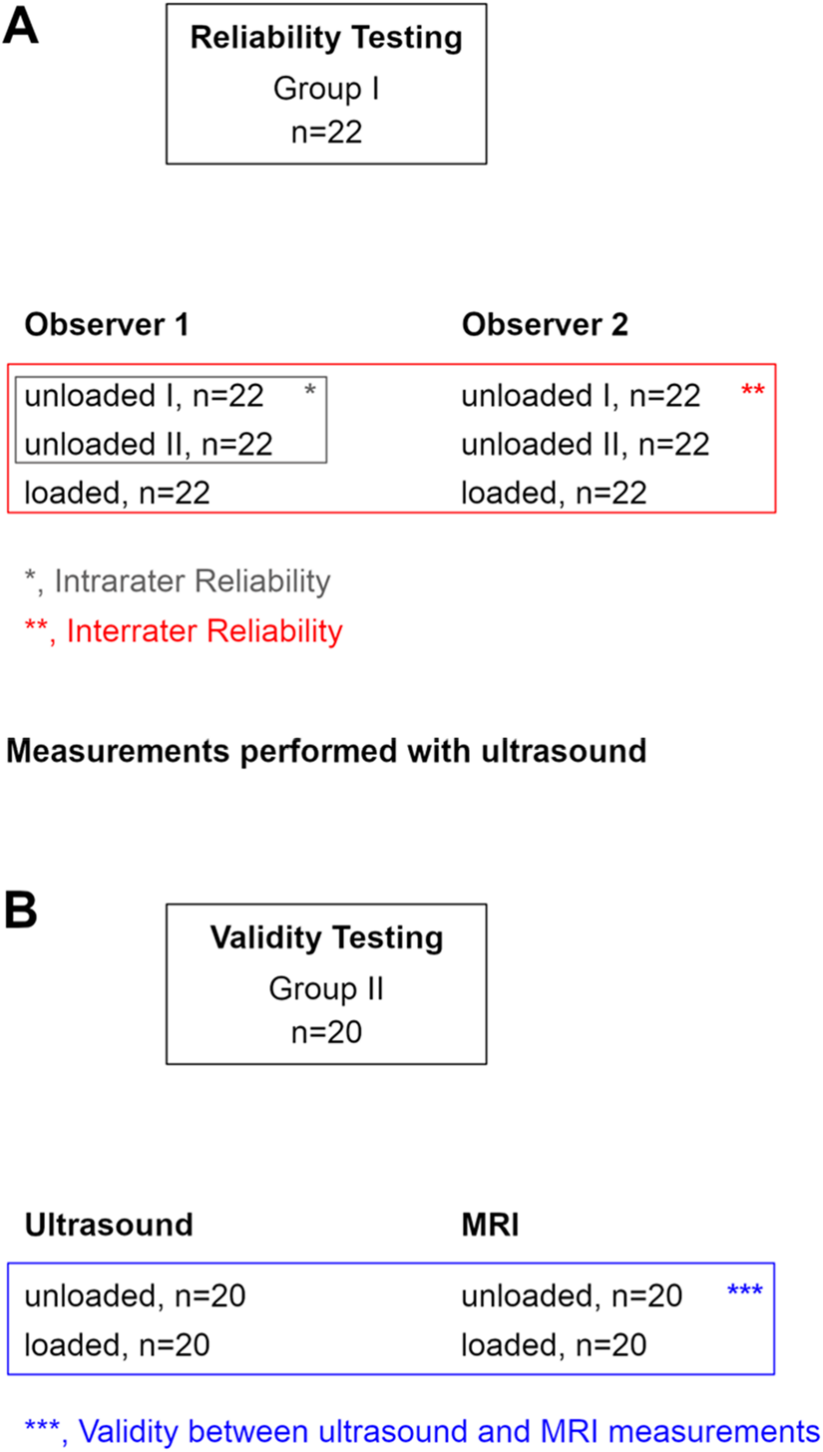
Group assignment. **a** Reliability testing based on Group I. Ultrasound (US) images were acquired twice in an unloaded condition and once in a loaded condition by each observer. Intrarater reliability testing was based on repeated measurements in the unloaded condition. Interrater reliability testing was based on measurements taken by each observer in the unloaded and loaded condition. “n” represents the number of images acquired by each observer in the respective loading condition. **b** Validity testing based on Group II. US and MRI images were acquired in both knees of all subjects in the unloaded and loaded condition. “*n*” represents the number of images acquired for each imaging modality in the respective loading condition

Patient recruitment, examination, and data acquisition for this prospective study were performed between May 2018 and May 2019. Eleven healthy volunteers (Group I; male and female) aged between 18 and 45 years were enrolled for reliability testing. Subjects in Group I had no history of knee injury or surgery and a standard clinical examination of the knee revealed no pathologic findings. Additionally, ten consecutive patients presenting for a follow-up (FU) examination (minimum 12 months FU) after anterior cruciate ligament reconstruction and all-inside lateral meniscal radial tear repair were asked for participation for validity testing (Group II). Lateral ME measurement is of high interest in patients undergoing radial meniscal tear repair since a correlation between lateral ME and radial meniscal tear healing is assumed [15]. Both knees were examined in all subjects, so that data of 22 and 20 knees were available in Group I and Group II, respectively (Fig. 1). In both groups, neutral leg alignment, clinically assessed using the methods described by Navali et al. [20] and Hinman et al. [21], was required for participation. Clinical signs of knee joint osteoarthritis [22, 23] as well as obesity (BMI ≥ 30) were criteria for exclusion. Lateral ME, measured under two loading conditions using US (Group I and Group II) and MRI (Group II only), served as the main outcome parameter.

Each subject was informed in detail about the rationale for and procedures involved in the study, prior to obtaining written and verbal consent for participation. Informed consent was obtained from each subject. The study was approved by the ethical review board of the Medical University of Innsbruck in February 2018 and registered in the European Union Clinical Trials Register (EudraCT-Number: 2017-005037-24).

### Radiological assessment

Image acquisition and analyses were performed by two observers (P.W.W., R.C.) in collaboration with a trained musculoskeletal radiologist (B.H.). Both knees were studied in the unloaded state and after application of axial load equivalent to 50% of the individuals’ body weight. Details of US and MR image acquisition and analyses are provided below.

### Ultrasound (US)

Sonographic evaluation of the postero-lateral meniscal corner was performed using a 5–10 MHz linear transducer in combination with the US unit Sonosite® MicroMaxx® (SonoSite, Inc., Bothell, WA, USA). Images were first acquired in the supine position (unloaded condition) and then in bipedal stance (loaded condition). 10° of knee flexion and 0° of tibia rotation were standard for examination. The optimal transducer position for ME measurement was located in the supine condition and marked to ensure consistent transducer positioning under both loading conditions. US images in the unloaded condition were acquired twice by each observer to determine test–retest reliability. US images in the loaded, standing condition were acquired once by each observer.

**Fig. 2:**
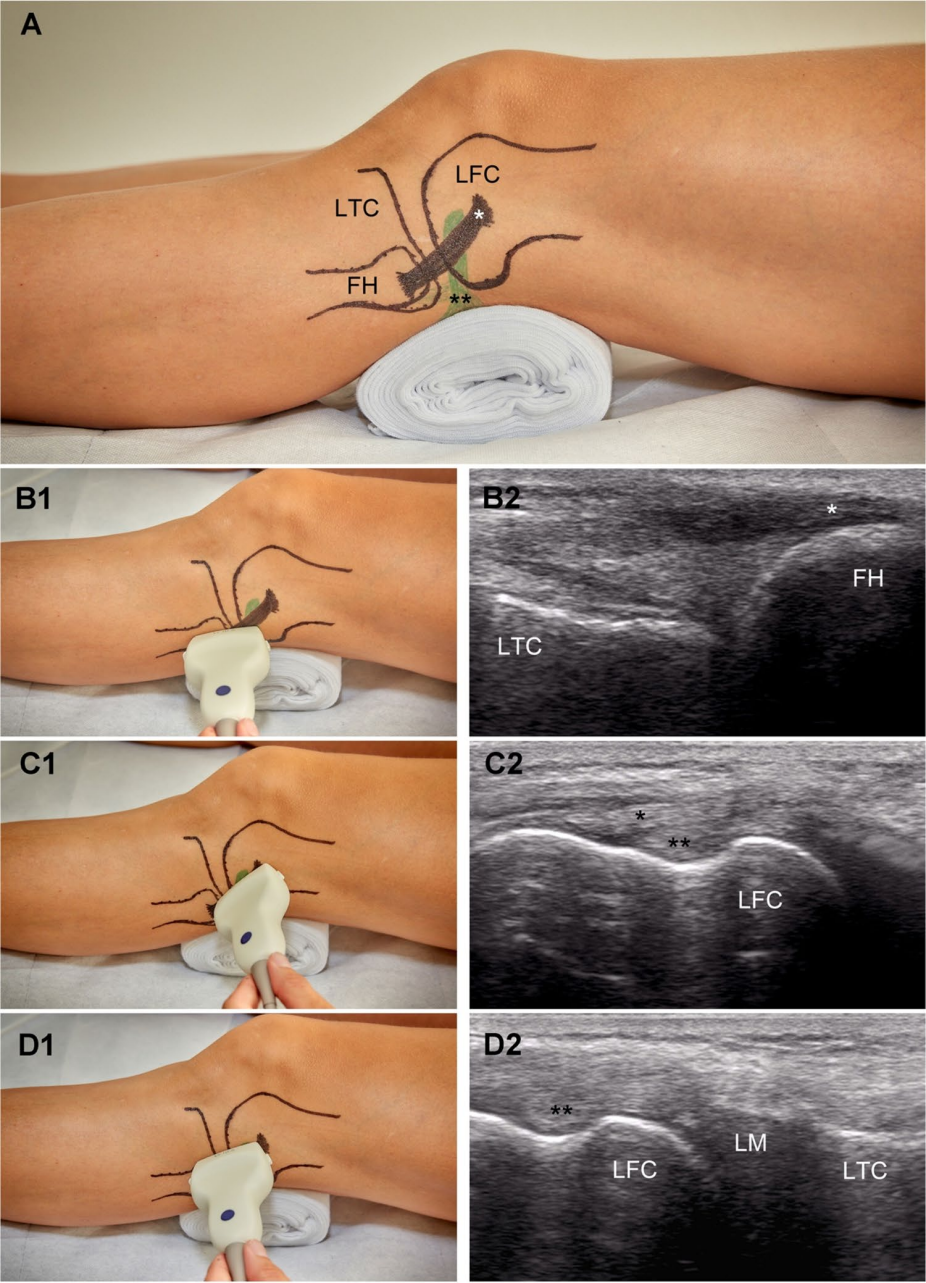
Standardized ultrasound-based examination of lateral meniscal extrusion. **a** Lateral view of the knee joint depicting the relevant landmarks. **b1**, **b2**, **c1**, **c2**, **d1**, **d2** Transducer positioning and related ultrasound image for slice 1, 2 and 3, respectively. FH, fibular head. LFC, lateral femoral condyle. *LM* lateral meniscus. *LTC* lateral tibial condyle. *, lateral collateral ligament. **, popliteus tendon

To our knowledge, no standardized US examination protocol for lateral ME measurements has been published yet. Therefore, the following approach was adopted to warrant consistency (Fig. 2). First, the fibula head and fibula attachment of the lateral collateral ligament (LCL) were located in the longitudinal plane. Then, the probe was shifted proximally along with the LCL until its femoral attachment appeared in the field of view. In this position, the probe was first pivoted to visualize the femoral origin of the popliteal tendon (PT) and then shifted centrally to the tibiofemoral joint line. With the probe held perpendicular to the joint plane and the tibial cortical rim, the orientation of the probe was slightly adjusted for optimal visualization of the distal lateral femur in combination with the PT, the proximal lateral tibial condyle, and the lateral meniscus in between. This final image was used for ME measurements.

### Magnetic resonance imaging (MRI)

MR images were recorded in both knees of the patients assigned to Group II in an unloaded and loaded condition. Standardized knee positioning and axial load application were achieved using an MRI-compatible, pneumatically driven knee brace device (Ergospect GmbH, Innsbruck, Austria), which has recently been described in detail [15]. Consistent with the US examinations, neutral rotational alignment of the lower leg and 10° of knee flexion were defined as the standard position for image acquisition. With subjects lying in the supine position, the unloaded images were recorded first. Then, axial pressure equivalent to 50% of the individuals' body weight was applied to simulate the bipedal stance.

A MAGNETOM Skyra 3.0 T MRI scanner (Siemens Healthcare AG, Erlangen, Germany) was used for image acquisition. Coronal, sagittal and axial fat-suppressed proton-density-weighted turbo spin-echo sequences were acquired for meniscus assessment in both loading conditions. The following MRI parameters were used: coronal (TR/TE = 4330/30 ms, FOV = 130 × 130 mm, slice thickness = 2 mm), sagittal (TR/TE = 2610/30 ms, FOV = 130 × 130 mm, slice thickness = 2 mm) and axial (TR/TE = 1950/29 ms, FOV = 150 × 150 mm, slice thickness = 2 mm). For lateral ME measurements, the coronal slice, in which the femoral origin of the PT was best visible, was selected.

### Image analyses

**Fig. 3:**
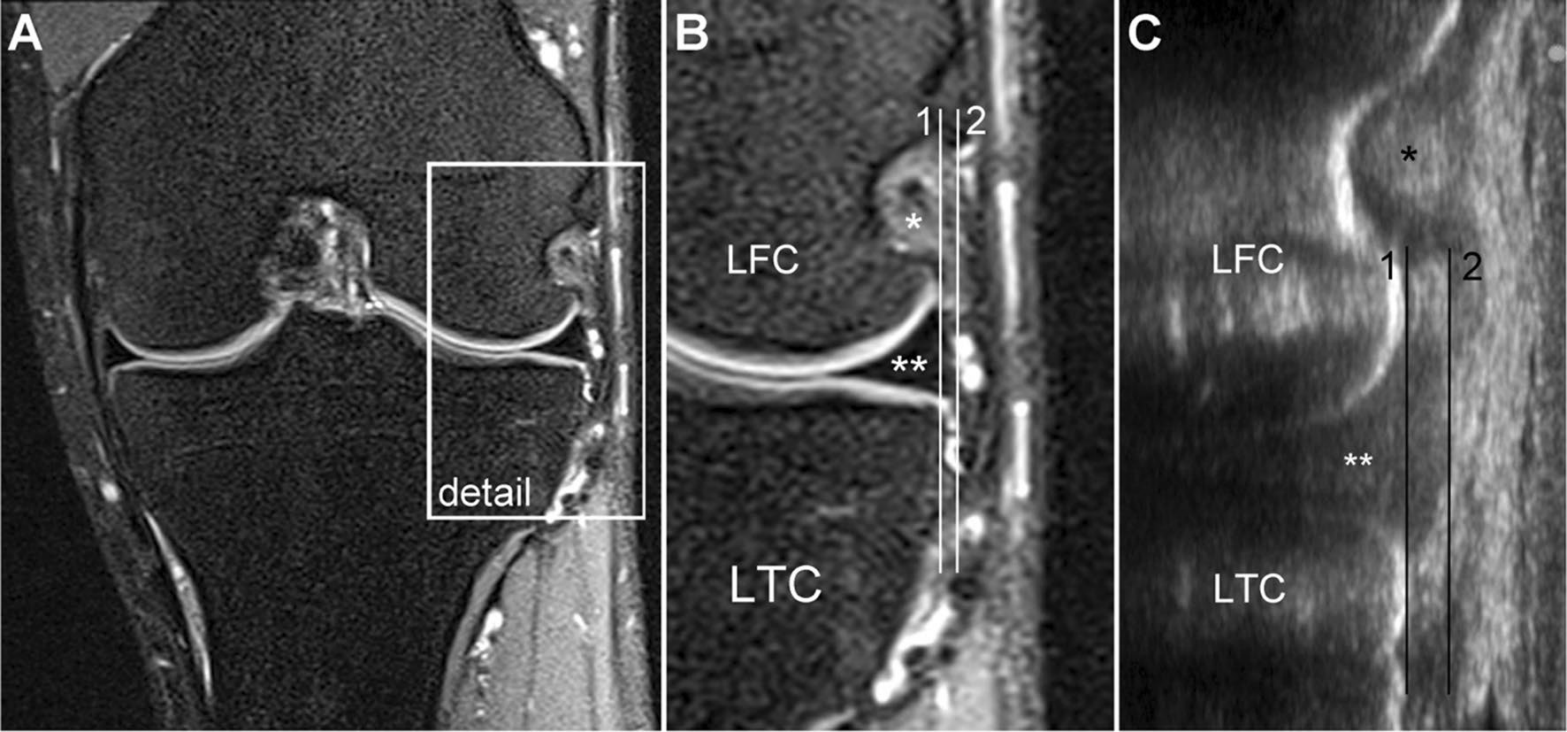
Measurement technique. **a** MRI overview. Detail of the MR image (**b**) and ultrasound slice (**c**) for meniscal extrusion measurement. Lateral meniscal extrusion is defined as the horizontal distance between line 1 and line 2. Line 1, running along the lateral tibial cortex margin. Line 2, running through the most peripheral lateral meniscus margin and parallel to line 1. *LFC* lateral femoral condyle. *LTC* lateral tibial condyle. *, popliteus tendon. **, lateral meniscus

Absolute lateral ME was defined as the horizontal distance between the lateral tibial cortex margin and the most peripheral border of the lateral meniscus. Analyses of US and MR images were performed using ImageJ version 1.52a (Wayne Rasband, National Institutes of Health, Bethesda, MD, USA) and Horos version 3.3.5 (https://horosproject.org, Annapolis, MD, USA), respectively. The precise procedure of image analyses is illustrated in Fig. 3.

### Statistical analyses

To assess interrater reliability for US examination, all measurements of lateral ME obtained by one examiner (means of two measurements obtained in the right and left knee in the supine, unloaded condition as well as the measurements obtained in the loaded condition) in Group I were pooled (total *n* = 44) and compared between the two raters by means of a paired samples *t* test. In addition, the intraclass correlation coefficient (ICC) was calculated using a two-way random-effects model to quantify the absolute agreement of measurements [24]. The typical error of measurement was calculated by dividing the standard deviation of difference scores by the square root of 2 [25]. A correlation plot was created for visual inspection of the agreement of ratings.

For test–retest (intrarater) reliability analysis, the test–retest data obtained by the main observer (P.W.W.) in the unloaded condition for Group I were compared and the intraclass correlation coefficient was calculated using a two-way mixed-effects model [24]. Further reliability statistics were calculated as for the interrater reliability analyses.

To assess measurement validity, the equivalence of US (*n* = 40) and MRI (*n* = 40) recordings of Group II was tested through the two one-sided *t* test (TOST) procedure for dependent means as per the principles of Lakens [26]. Differences of 0.5 mm were defined as the upper and lower limits of clinical equivalence. In addition, the ICC and typical error of measurement were calculated as for the test of intrarater reliability.

Additionally, group comparison (unloaded vs. loaded) for both imaging modalities for each group was performed by a paired t test. A paired t-test was also used to compare the healthy knee and the surgically treated knee in Group II (healthy vs. injured). Normal distribution of all data was confirmed using the Shapiro–Wilk test. Statistical analyses were performed using IBM SPSS Statistics (version 25, IBM, Armonk, NY, USA) and R (version 3.6.1, R Core Team, Vienna, Austria).

## Results

Eleven subjects (9 males, 2 females) with a mean age of 25.5 ± 3.75 years (18–45 years) were included in Group I. For Group II, ten patients (8 males, 2 females) with a mean age of 30.0 ± 6.45 years (23–43 years) were enrolled. All participants were able to complete all examinations, thus, no secondary exclusions were required.

## Reliability testing

### Interrater reliability

**Fig. 4:**
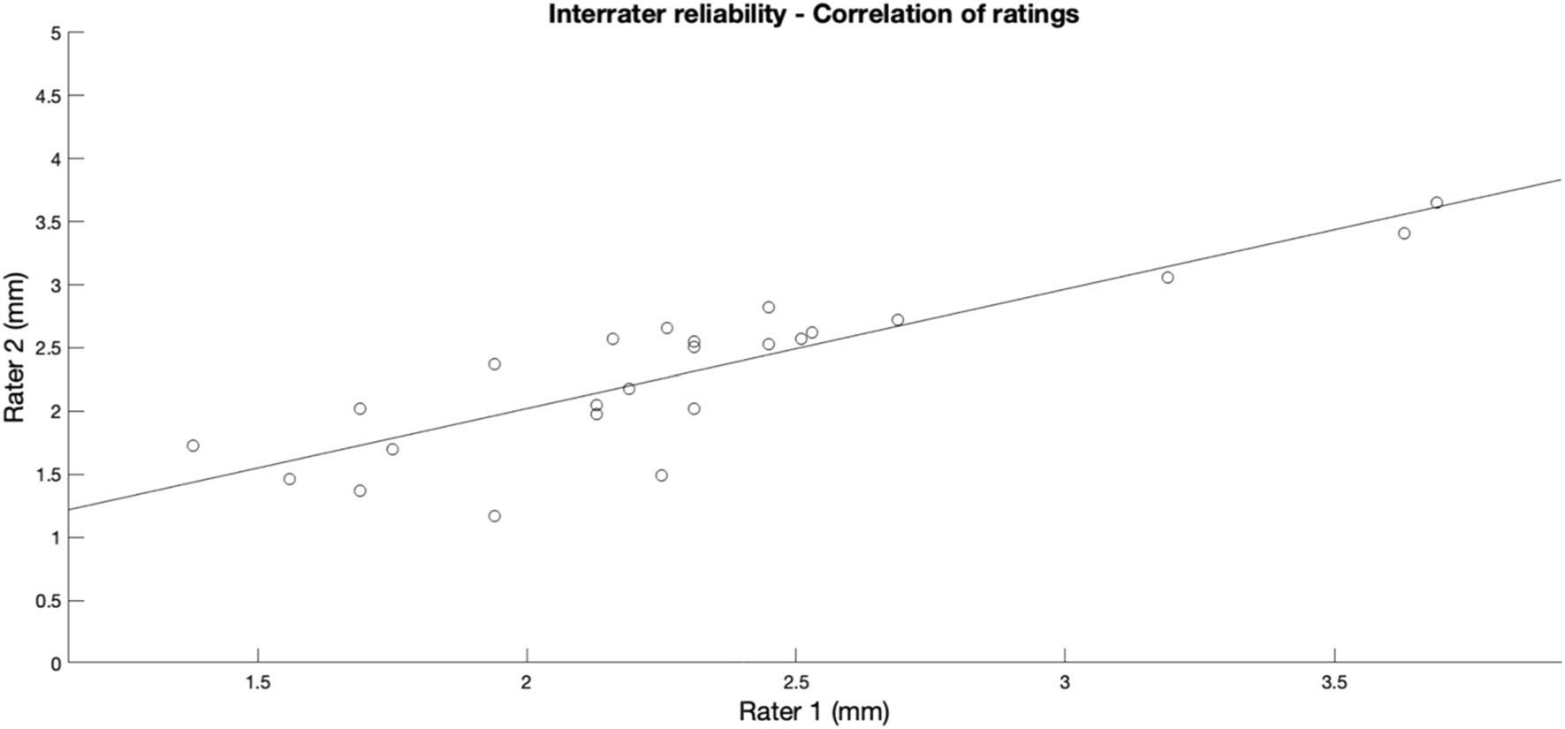
Correlation plot of interrater reliability for ultrasound-based measurements of lateral meniscal extrusion

The average US-based measurements of lateral ME obtained by the two observers for Group I were 2.5 ± 0.6 mm and 2.5 ± 0.7 mm, respectively. This difference was found to be non-significant (*t*(43) = − 0.695, *p* = 0.491). The intraclass correlation coefficient testified to good agreement of ratings (ICC = 0.904, 95% CI: 0.824–0.947). The typical error of measurement was 0.2 mm. A correlation plot of interrater reliability is shown in Fig. 4.

### Intrarater reliability

**Fig. 5:**
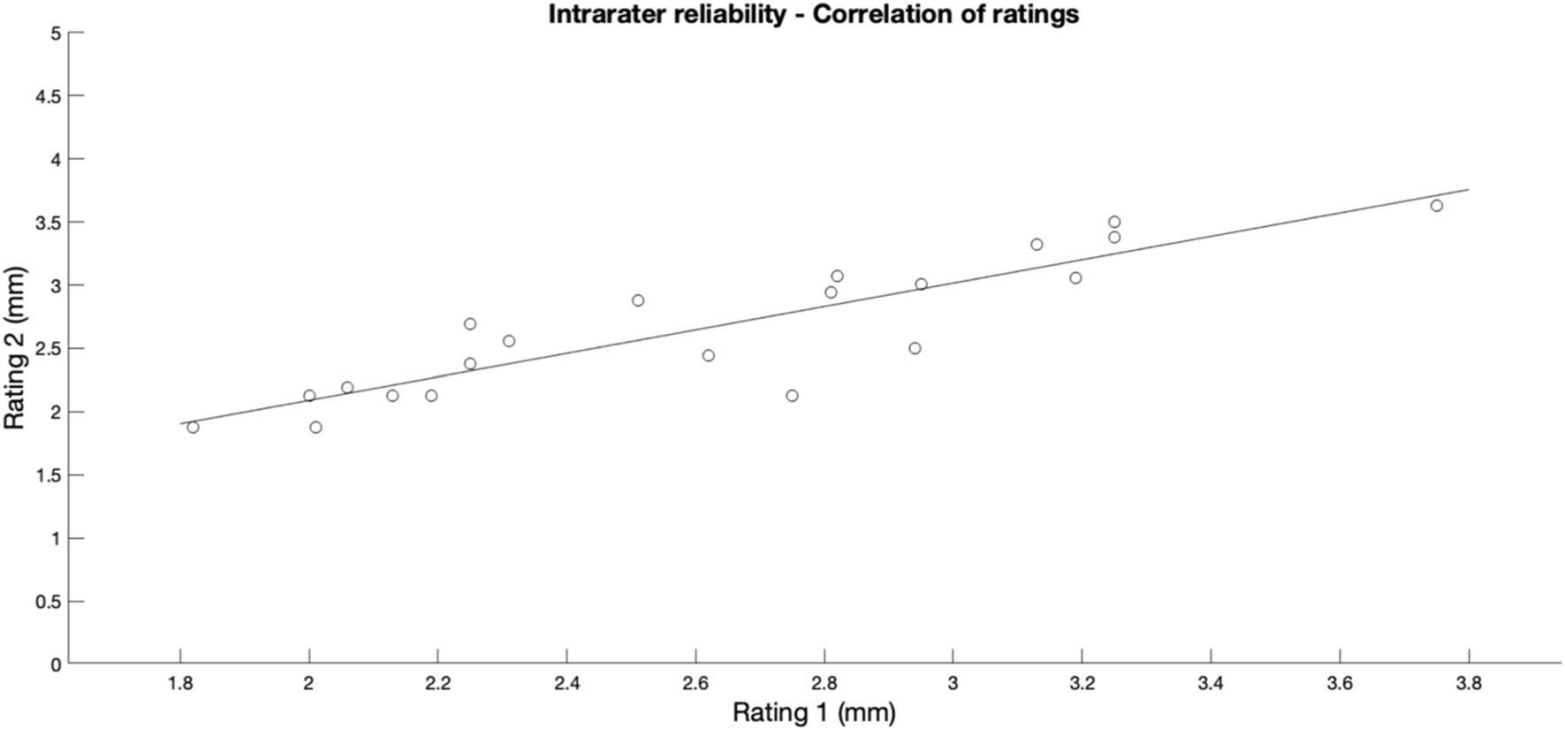
Correlation plot of intrarater (main observer 1) reliability for ultrasound-based measurements of lateral meniscal extrusion

The data obtained through repeated US-based measurements in the supine position (unloaded condition) for Group I were very similar and not statistically different (2.6 ± 0.5 mm vs. 2.7 ± 0.5 mm; *t*(21) = − 0.837, *p* = 0.412). Excellent reproducibility of measurements was also confirmed by the intraclass correlation coefficient of 0.942 (95% CI: 0.861–0.976) and the typical error of measurement was 0.2 mm. A correlation plot of interrater reliability is shown in Fig. 5.

### Validity testing

**Fig. 6:**
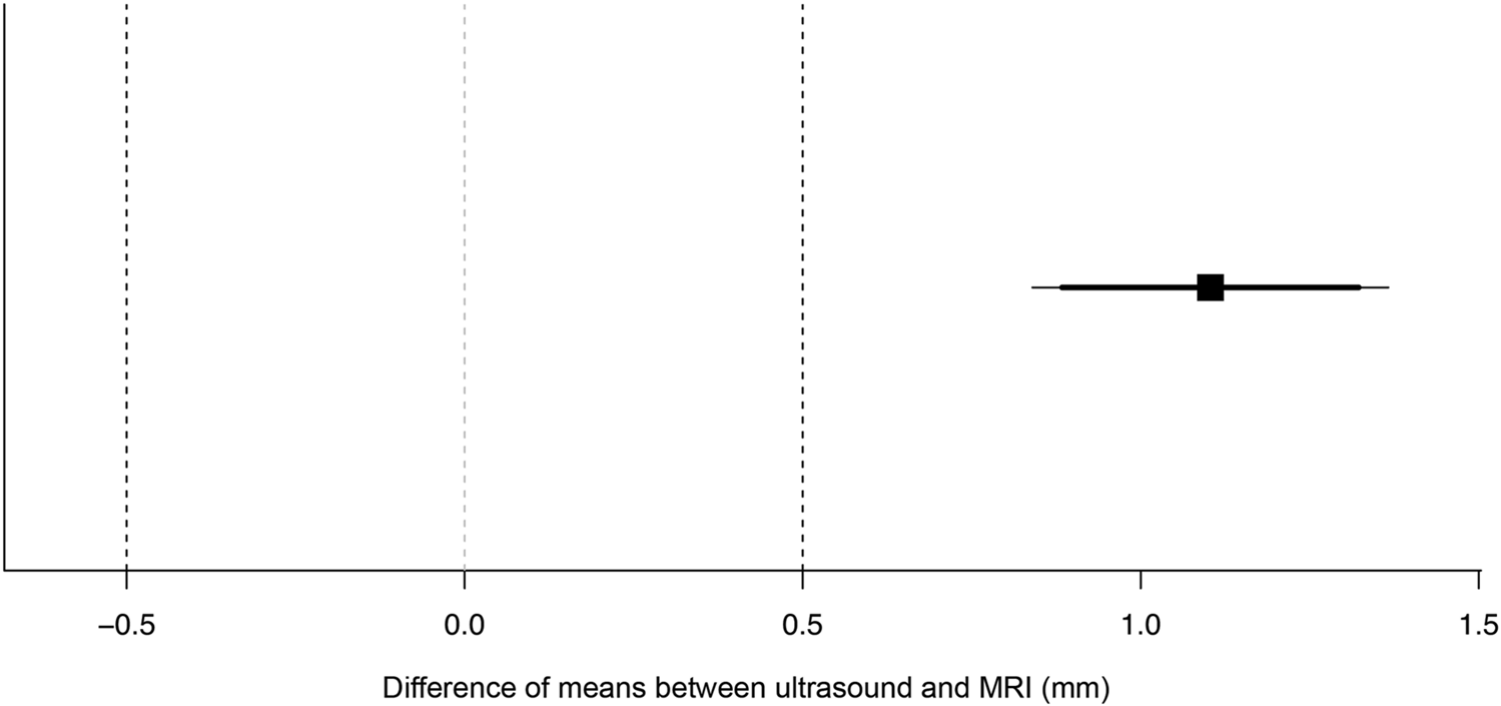
Two one-sided* t* test (TOST) of equivalence of MRI and ultrasound recordings. Lateral meniscal extrusion as measured by ultrasound is greater by 1.1 mm as compared to MRI results. Measurements are not within the defined bounds (0.5 mm) of equivalence

**Fig. 7:**
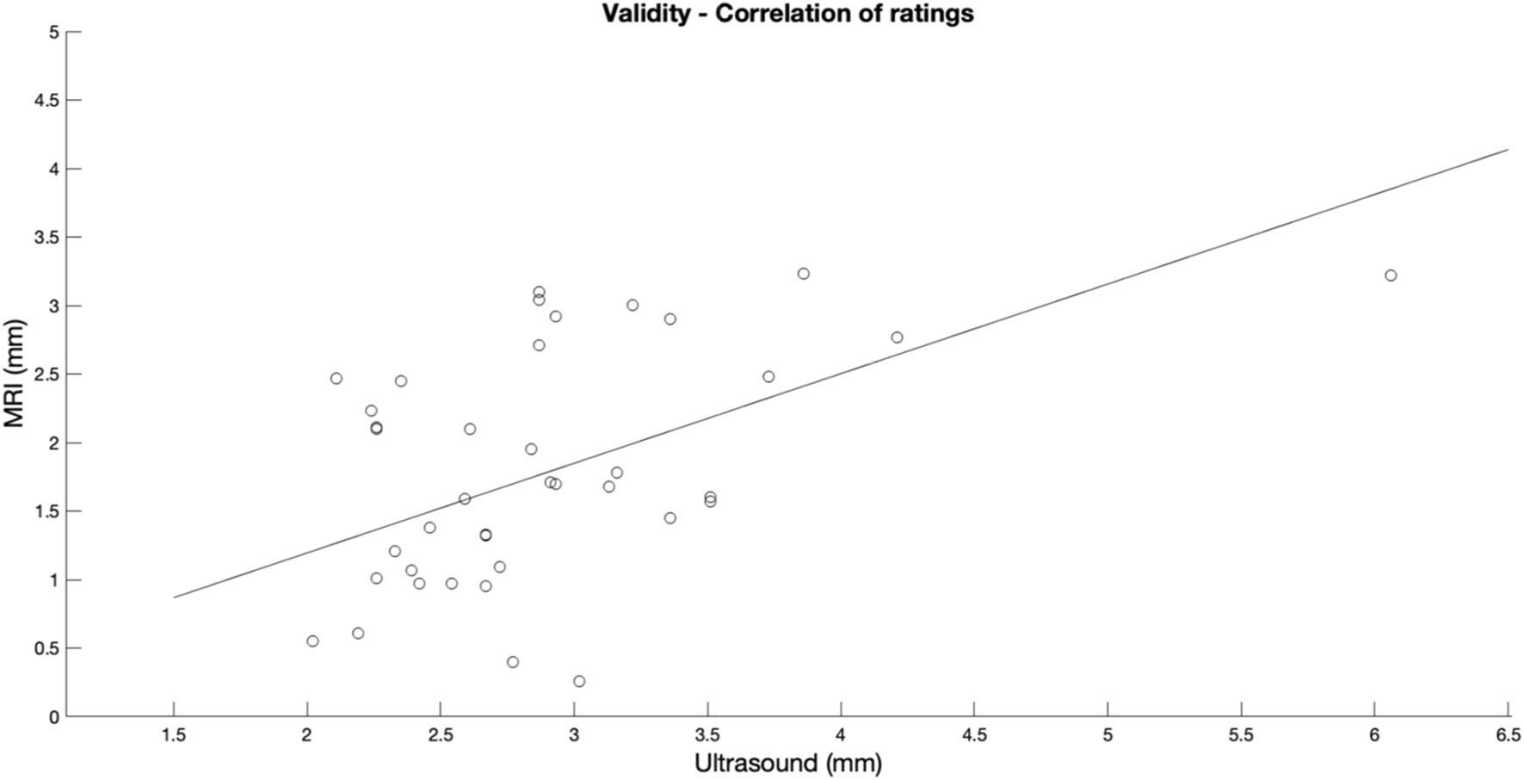
Correlation plot to visualize the agreement of MRI- and ultrasound-based measurements of lateral meniscal extrusion

In Group II, the mean lateral ME for both loading conditions as measured by US and MRI were 2.9 ± 0.8 mm and 1.8 ± 0.9 mm, respectively. The comparison of data revealed that ME as measured by US was greater by 1.1 mm (61.1%) as compared to MRI. Accordingly, the results of the TOST procedure confirmed that the means were not within the equivalence bounds of 0.5 mm (*t*(39) = 4.633, *p* = 1.000). The systematic overestimation of lateral ME by US is evident from Fig. 6. The intraclass correlation coefficient reflected poor agreement of measurements (ICC = 0.439, 95% CI: − 0.221 to 0.750) and the typical error of measurement was 0.6 mm. A correlation plot reflecting the agreement of MRI and US data is shown in Fig. 7.

### Group comparison

In Group II, more lateral ME could be observed in the loaded compared to the unloaded condition (Table 1). This difference was statistically significant for MRI-based measurements (*t*(19) = 2.110, *p* = 0.048), while no significant difference was observed for US-based measurements (*t*(19) = 0.353, *p* = 0.728). Interestingly, in Group I, the lateral ME as assessed by US was significantly smaller in the loaded condition compared to the unloaded condition, *t*(21) = − 4.536, *p* < 0.001. Comparison of the healthy and the surgically treated (injured) knees in Group II showed statistically significantly greater lateral ME for the surgically treated (injured) knees compared to the healthy knees in both MRI- and US-based measurements (MRI, *t*(19) = 6.583, *p* < 0.001; US, *t*(19) = 2.507, *p* = 0.021; Table 2).

**Table 1:** Lateral meniscal extrusion for the different loading conditions

	Group I (*n* = 22)			Group II (*n* = 20)					
	Ultrasound			Ultrasound			MRI		
	Unloaded	Loaded	*p* value	Unloaded	Loaded	*p* value	Unloaded	Loaded	*p* value
Lateral ME [mm]	2.6 ± 0.5	2.2 ± 0.5	< 0.001*	2.8 ± 0.5	2.9 ± 1.0	0.728	1.6 ± 0.8	1.9 ± 1.0	0.048*

Measurements of lateral meniscal extrusion are presented as mean ± standard deviation

*ME* meniscal extrusion; *n* number of investigated knees for each group and each loading condition

*Statistically significant difference between the unloaded and loaded condition (*p* < 0.05)

**Table 2:** Lateral meniscal extrusion in the healthy and surgically treated (injured) knees in Group II

	Ultrasound		*p* value	MRI		*p* value
	Healthy (*n* = 10)	Injured (*n* = 10)		Healthy (*n* = 10)	Injured (*n* = 10)	
Lateral ME [mm]	2.6 ± 0.5	3.1 ± 0.9	0.021*	1.2 ± 0.7	2.3 ± 0.7	< 0.001*

Measurements of lateral meniscal extrusion are presented as mean ± standard deviation

*ME* meniscal extrusion; *n* number of investigated knees

*Statistically significant difference between the healthy and surgically treated (injured) knees (*p* < 0.05)

## Discussion

The main findings of the present study were that US-based measurement of lateral ME (1) yields highly reliable data, provided a standardized measurement protocol is used, but (2) overestimates lateral ME as compared to MRI measurements, resulting in the poor agreement of measurement results.

Magnetic resonance imaging, the gold standard for meniscus evaluation [14], is time-consuming, costly and limited in that the acquisition of stress images [6, 11, 15] is not possible unless sophisticated loading devices are used. US, by contrast, is cost-effective, readily available and easily applicable in the standing position to obtain images under axial knee compression. However, while the technique has been successfully used for measurements of medial ME [3, 16–18], the validity and reliability of US-based measurements of lateral ME are unknown. In the present study, in vivo data of lateral ME analyzed by US were compared to data obtained by MRI.

Under load-bearing conditions, the menisci cover 59–71% of the tibial plateau articular surface [27]. This leads to an increase of the tibio-femoral contact area and a reduction of the transmitted contact pressure, thus protecting the cartilage from excessive stress and preventing early joint degeneration [1]. Meniscus tears are associated with increased ME [7, 8, 12, 19], leading to a decreased contact area and, consequently, increased contact pressure. This may ultimately promote the development or progression of knee joint osteoarthritis [9, 28]. In the present study, lateral ME was significantly higher in surgically treated knees compared to the contralateral healthy knees. This indicates that a combined anterior cruciate ligament reconstruction and lateral meniscal radial tear repair is not able to restore lateral ME to the level of the healthy contralateral knee. Thus, ME is an indicator for various pathologies [7, 8, 12] and can be used as a screening tool for knee joint osteoarthritis [10, 11, 29]. Additionally, recent studies [30, 31] have demonstrated that ME represents a negative prognostic factor regarding the outcome of arthroscopic partial meniscectomy. Considering the increasing clinical interest in ME, measurements need to be simple, reliable and cost-effective. In one study, 63% of patients with Schatzker type IV tibial plateau fractures had concomitant lateral meniscal injuries diagnosed by MRI [32]. The authors assumed that the incidence of true lateral meniscal injuries was overestimated by MRI [32]. Therefore, US-based assessment of lateral ME may be used to evaluate the functional integrity of the lateral meniscus in Schatzker Type IV fractures to facilitate treatment decision-making regarding the meniscus.

Unexpectedly, higher values of lateral ME were observed in the unloaded compared to the loaded condition in Group I, while no significant difference was found in Group II. Given the physiological varus alignment of the lower limb and the positive correlation between medial ME and varus alignment [33, 34], an inverse effect for the lateral meniscus under loading conditions may be an explanation for this observation.

Numerous studies [2, 3, 9, 10, 13, 17, 29] have investigated medial ME using US. To obtain reliable measurements, distinct landmarks were defined, including the medial femoral epicondyle, the medial tibial condyle and the three-layered medial collateral ligament. To achieve optimal visualization of medial ME, the transducer is aligned in a longitudinal direction parallel to the fibers of the medial collateral ligament. As opposed to the detailed recommendations for the US-based examination of medial ME, scant literature about lateral ME measurements is available [19, 35, 36]. Rowland et al. [19] and Verdonk et al. [36] placed the longitudinally oriented transducer just anterior to the LCL but did not consider any further anatomical landmarks. Riecke et al. [35] did not provide information on the positioning of the transducer.

The present study represents the first attempt to provide a standardized US protocol for reliable in vivo investigation of lateral ME. Despite the known examiner-dependency of US, usage of clearly identifiable landmarks of the postero-lateral knee corner and consistent imaging planes allowed for ME to be measured with good interrater (ICC = 0.904) and excellent intrarater (ICC = 0.942) reliability.

Different techniques for ME measurement have been described in the literature [5, 37, 38]. Coronal MR slices, as obtained in the present study, are oriented parallel to the posterior condylar line of the femur and represent the imaging standard for measurements of ME. It should be noted, however, that strictly coronal images may lead to both underestimations of ME, when menisci extrude anteriorly or posteriorly from the imaging plane, and overestimations of ME, when measurements are obtained posterior to the joint midline. For these reasons, Jones et al. [38] have recently recommended obtaining radially orientated MR slices, running perpendicular to the tibial and meniscal rim, to minimize the risk of bias due to incorrect slice angulation. Since the spatial resolution of both MRI and US is excellent, deviations of results are likely due to differences in imaging planes. In the present study, US measurements of lateral ME were greater by 1.1 mm (+ 61.1%) as compared to extrusion measurements by MRI, resulting in the poor agreement of results. Thus, absolute extrusion measurements are not readily comparable to MRI. Nevertheless, reliability tests have demonstrated that US measurements of lateral ME are highly reproducible both within and between examiners, suggesting that the technique may be used to evaluate ME, provided that results are compared to US-specific normative data. In addition, usage of US appears feasible to longitudinally track the progression over time. Current literature reflects the increasing importance of evaluation of dynamic ME, [3, 12, 13] and underlines the necessity to develop cheap, simple and readily-available alternatives to MRI for its assessment.

## Limitations

Some limitations have to be considered when interpreting this study. First, patients presenting with clinical signs of knee joint osteoarthritis [22, 23] were excluded from this study. Therefore, reliability was only confirmed for knees free of joint degeneration. Osteoarthritis is accompanied by synovial hypertrophy [39], osteophytes and increased ME [2, 5, 9–11], potentially hindering reliable US assessment. However, no MRI examination was performed in the healthy volunteers assigned to Group I. The inclusion was based on the patients’ medical history and clinical examination. Consequently, existing asymptomatic degenerative changes to the lateral meniscus could not be excluded, which may have affected the results.

An increasing amount of subcutaneous fatty tissue requires an increased US penetration depth. This leads to a lower resolution [40], complicating meniscus assessment and accurate extrusion measurement. In the present study, a BMI smaller than 30 was necessary for enrollment, suggesting that the presence of excessive perigenicular fatty tissue is unlikely. The assessment of lateral ME in patients with a BMI greater than 30 may be more challenging.

The examination of both knees under two different loading conditions resulted in a high number of images available for ME measurement. However, the absolute number of participants is a limiting factor of this trial.

## Conclusions

In summary, the present study provides evidence that:

- The implementation of a standardized US-based examination protocol leads to high levels of reliability for lateral ME measurement.
- Agreement with MRI, the current gold standard for meniscal extrusion measurement, was poor, with US systematically overestimating MRI results by 1.1 mm on average.

Based on these findings, we conclude that US is a reliable technique for lateral ME measurement, but measurement results are not readily comparable to MRI.

## Data Availability

The datasets used and analyzed during the current study are available from the corresponding author on reasonable request.

## Abbreviations

FU: Follow-up
ICC: Intraclass correlation coefficient
LCL: Lateral collateral ligament
ME: Meniscal extrusion
MRI: Magnetic resonance imaging
PT: Popliteal tendon
TOST: Two one-sided *t* test
US: Ultrasound
